# Women's Attitudes towards the Option to Choose between Karyotyping and Rapid Targeted Testing during Pregnancy

**DOI:** 10.1155/2013/636459

**Published:** 2013-04-30

**Authors:** Angelique J. A. Kooper, Dominique F. C. M. Smeets, Ilse Feenstra, Lia D. E. Wijnberger, Robbert J. P. Rijnders, Rik W. P. Quartero, Peter F. Boekkooi, John M. G. van Vugt, Arie P. T. Smits

**Affiliations:** ^1^Department of Human Genetics, Radboud University Medical Centre, P.O. Box 9101, 6500 HB Nijmegen, The Netherlands; ^2^Department of Obstetrics and Gynaecology, Rijnstate Hospital, 6815 AD Arnhem, The Netherlands; ^3^Department of Obstetrics and Gynaecology, Jeroen Bosch Hospital, 5200 ME ‘s-Hertogenbosch, The Netherlands; ^4^Department of Obstetrics and Gynaecology, Medical Spectrum Twente, 7500 KA Enschede, The Netherlands; ^5^Department of Obstetrics and Gynaecology, St. Elisabeth Hospital, 5022 GC Tilburg, The Netherlands; ^6^Department of Obstetrics and Gynaecology, Radboud University Medical Centre, 6500 HB Nijmegen, The Netherlands

## Abstract

*Objectives*. Pregnant women, referred because of an increased risk of fetal Down syndrome, who underwent an invasive prenatal procedure were offered a choice between karyotyping and rapid targeted testing. This study aims to assess women's attitudes and experiences towards what option to choose. *Methods*. A retrospective multicentre survey (2008–2010) was conducted among 1370 women. General questions were asked about decision making issues, followed by personal questions about their experiences in choice making, test preference, influence of others, and possible regrets. *Results*. In total, 90.1% of the respondents (*N* = 825) indicated that pregnant women are able to choose, although 33.1% stated that the choice can best be made by a professional. 18.4% indicated that making a choice places a burden on women. In 96.4%, respondents preferred to have the option to choose again in case of a next pregnancy, whereas 2.7% preferred the choice to be made by a professional. Regret was indicated by 1.2%. Decision making was influenced by others in 64.9%. A slightly higher preference for karyotyping was indicated by 52.7% of the respondents. *Conclusions*. Positive attitudes and experiences were expressed towards the option to choose. Respondents took decisions freely, although sometimes influenced by a partner or a professional, to follow their individual perspectives.

## 1. Introduction

 Currently, in many prenatal centres, rapid targeted tests (QF-PCR: quantitative fluorescent polymerase chain reaction or MLPA: multiplex ligation-dependent probe amplification) are used for the prenatal detection of common chromosomal aneuploidies. The need for rapid testing methods, not requiring cell culture, has been recognized in order to improve pregnancy management and to alleviate parental anxiety [1, 2]. Analysis of large series of prenatal samples by QF-PCR or MLPA has shown that both tests, deliberately targeted to the analysis of selected chromosomes, that is, chromosomes 13, 18, and 21 with or without inclusion of the sex chromosomes, allow the detection of the vast majority of chromosome abnormalities in prenatal samples [3–9].

 The application of such targeted tests as stand-alone tests is still strongly debated in various countries, and therefore, different approaches are presently applied. In some centers, women with a risk of having a fetus with a chromosomal abnormality (without fetal ultrasound anomalies) will only be offered a rapid targeted test, whereas in other centres, they can choose between a rapid targeted test and karyotyping or, alternatively, are offered routine karyotyping only. 

 In the last decades, patient's individual views, values, and wishes have gradually become more important. Personal autonomy and individualized choice have increasingly driven patient's autonomy to the forefront of health care [10], particularly in prenatal testing [11]. In this regard freedom of choice as to what test should be applied appears to be of interest. So far, however, the question remains: what is the patients' experience?

 During this study in our prenatal diagnostic centre, the indications for prenatal testing were divided in seven main indication groups (1)–(7) and examined with different types of diagnostic tests (I)–(IV).

- A choice between karyotyping and rapid targeted testing was offered to women with (1) advanced maternal age (1) or to women with a risk > 1 : 250 following Down syndrome screening using the first trimester combined test (2).
- Rapid targeted testing only (targets chromosomes 13, 18, 21, X, and Y) was offered when invasive testing was performed for molecular or biochemical testing for a genetic disorder (3).
- Karyotyping was performed when there are known familial chromosome rearrangements, for example, Robertsonian or reciprocal translocations (4) or a known or suspected family history of chromosome abnormality (5).
- Genome-wide microarray analysis (after normal rapid targeted test) was performed when there is an ultrasound detection of any major structural abnormality (including nuchal translucency > 3.5 mm before 14-week gestation) (6) or whether there is a previous child with a submicroscopic aberration (7).

During the last four years, five hospitals within our prenatal service program have offered pregnant women, referred for an amniocentesis or chorionic villus sample (CVS) because of advanced maternal age (maternal age ≥ 36 years) or a risk > 1 : 250 following Down syndrome screening using the first trimester combined test, the option to choose between a rapid targeted test and karyotyping as stand-alone tests. The aim of the present study was to evaluate pregnant women's perspective and satisfaction towards the option to choose between these tests.

## 2. Material and Methods

 This retrospective survey involved the collection of information from respondents by use of an anonymous questionnaire. After a pilot test with 50 questionnaires, the final questionnaire was sent out in April 2011 to 1370 women who underwent an invasive procedure in the period from March 2008 till December 2010 to collect as many respondents as possible. The reason for amniocentesis or CVS was advanced maternal age and/or an increased risk at first trimester combined test (nuchal translucency (NT) < 3.5 mm). The combined test comprised three markers, that is, maternal serum beta human chorionic gonadotropin (beta-hCG), maternal serum pregnancy-associated plasma protein-A (PAPP-A), and ultrasound measurement of NT. These three markers together with maternal age provided a patient-specific risk. Women with a fetal NT > 3.5 mm were not included. A fetal NT > 3.5 mm can be associated with structural malformations, other genetic syndromes, or submicroscopic aberrations and examined best by genome-wide testing (e.g., by microarray analysis). 

 Women were from hospitals participating in the Network Prenatal Diagnostics Nijmegen (NPDN) consisting of the Radboud University Medical Centre (hospital A) and four satellite clinics, that is, Rijnstate Hospital Arnhem (hospital B), St. Elisabeth Hospital and TweeSteden Hospital Tilburg (hospital C), Medical Spectrum Twente Enschede (hospital D), and Jeroen Bosch Hospital's Hertogenbosch (hospital E), all following the same standards of prenatal diagnostic care and covering the southeast part of The Netherlands.

After making an appointment for invasive testing, written information was sent to the women. In all hospitals, women were offered a prenatal counseling although counseling varied in time and type of professional. In hospitals A and E, the genetic counseling session generally lasted about 30 minutes and was performed by a genetic counselor or medical doctor, respectively. In hospitals B (medical doctor), C (genetic counselor), and D (medical doctor), this procedure lasted about 5 minutes. 

 In 1332 of the cases (97.3%), the women received a normal test result on their amniocentesis or CVS, and in 38 of the cases (2.7%) the women received an abnormal test result (trisomy 21 *N* = 31; trisomy 18 *N* = 1; sex chromosomal aberration *N* = 6). These latter women were asked if they would be willing to participate in the study before the questionnaire was sent out (nonanonymous). 

 A 10-question self-designed questionnaire was used to collect data on satisfaction with having the option to choose between the two types of diagnostic tests. The questions were categorized into three groups: (a) personal characteristics (age, education level, test of choice, and prenatal centre), (b) general attitudes towards having a choice, and (c) questions about the personal experience with having a choice. Category (b) comprised four questions, each with a 5-point rating scale ranging from strongly disagree to strongly agree. Category (c) comprised closed-ended questions with the possibility to answer yes or no, however, with the option to add a personal comment. Questionnaires had to be returned within six months. The data was analyzed using a Statistical Package for the Social Sciences (SPSS) software package. Chi-square tests were used to determine if there were statistically significant differences between responses (*P* < 0.05). 

## 3. Results

Eight hundred and twenty-five (825) completed surveys were returned. Thirty-five of the 1370 surveys were sent out to a wrong address, leaving a total evaluable sample of 1335, resulting in a response rate of 61.8%.

There were 38 women who had received an abnormal fetal test result. Only 12 out of these 38 (31.6%) responded (trisomy 21 *N* = 8; trisomy 18 *N* = 1; 47, XXY *N* = 2; mosaic 45, X/46, XY *N* = 1), which is much lower than the overall response. Not all responding women answered all survey items, which resulted in a variable number of respondents per question. The mean maternal age of the respondents was 38.0 ± 2.8 years (mean ± standard deviation). Just over half (55.0%) followed a higher professional education or university. In 52.7%, the test of preference was karyotyping, 40.5% of the respondents chose to have a rapid targeted test, and 5.6% did not remember the type of test they underwent. The respondents were women recruited from 5 different locations: hospital A (35.4%), hospital B (22.7%), hospital C (11.4%), hospital D (11.6%), and hospital E (18.9%). An overview of all personal characteristics is shown in Table 1. 

### 3.1. Respondent's General Attitude about Having the Option to Choose

Almost all respondents (97.9%) agreed that pregnant women should have the possibility to choose between tests, and 90.1% indicated that pregnant women are able to choose between both tests. Nevertheless, one-third of the respondents (33.1%) suggested that the choice can best be made by a professional. In 18.2%, choosing between tests was experienced as a burden to pregnant women. An overview of all respondents' general attitude about having a choice in prenatal testing is shown in Table 2.

### 3.2. Respondent's Personal Experience about Having the Option to Choose

Regret was indicated by ten (1.2%) women: in six a rapid targeted test was the test of choice, three chose karyotyping, and one did not remember which test was done. Comments from these respondents are shown in Table 3. In two cases, the test of choice could not be performed because of technical reasons (case id 59 and 463). In two cases (case id 266 and 725), detection of a fetal heart defect during pregnancy followup at a later gestational age was the reason for regret. They had chosen a rapid targeted test, while the cardiac defect could be related to a (small) cytogenetic aberration not detected with this test. The other four respondents reported different personal reasons to have regrets: the time to decide was too short; doubt, inability to make a choice, and familiarity with karyotyping in a previous pregnancy were the aspects they mentioned. 

Three other pregnancies ended in fetal demise. These respondents also showed regret but not because of the choice they made but because they underwent the invasive procedure. Two of them did not have an invasive procedure in a following pregnancy. Three of the four women who received an abnormal diagnostic test result and chose to have a rapid targeted test reported that they were positive about having the test result so quickly. 

Although decision making was influenced by others in 64.9% of the women (influence of partner 28.8%, professional 10.4%, and combination of partner, professional, and others 25.7%), most of the women (96.2%) felt they made the choice freely and were able to follow their personal perspectives.

In a next pregnancy, 2.7% of the respondents preferred to have the choice to be made by the professional; however, the majority (96.8%) wanted to have the possibility to choose again. An overview of all respondents' personal experiences about having a choice in prenatal testing is shown in Table 4.

On the basis of the maternal age distribution, the mean maternal age of women preferring a rapid targeted test or karyotyping was 37.7 years ± 3.03 SD and 38.3 years ± 2.60 SD, respectively. Karyotyping appeared the preferred test in the group with advanced maternal age (48.5%) compared to women younger than 36 years (34.5%). 

Regarding the respondents' test of preference, we observed considerable and statistically significant differences between hospitals A and E (Table 5). In hospital A, 31.2% preferred a rapid targeted test, and 63.0% chose to have karyotyping, while in hospital E the opposite was the case: 60.1% and 32.7%, respectively (*P* < 0.001, chi-square test). Examination of both populations showed no differences in the level of education or in having regret of their choice. Differences were seen in age distribution and influence of others: the population in hospital E comprised more respondents <36 years (*P* = 0.004). Although both hospitals A and E apply a 30-minute counseling regime, the influence of others on choicemaking was higher in hospital E (*P* = 0.025). Nevertheless, women from both hospitals appeared positive about their choice.

## 4. Discussion

 Our results show that more than 90% of the pregnant women who answered the questionnaire expressed a positive attitude towards the option to choose between karyotyping and rapid targeted testing. Although most of the women (64.9%) felt influenced by their physicians and their partners, the majority (96.2%) had the impression that they were free to choose and to follow their individual perspectives. The question of whether the option to choose places a burden to women was answered positively in 18.4% of the respondents; however, almost all women in our cohort (96.4%) preferred to have the option to choose again in a next pregnancy. 

 Ethical discussions about the desirability of offering different prenatal tests focus mainly on the autonomy of the women involved. A growing consensus is that women will only benefit from the test offer when they are able to make an autonomous decision [12]. Our study shows that most women, although influenced by others, are positive about choice making. In 50% and 46.1%, respectively, respondents with a lower educational or middle education level preferred the choice to be best made by a professional, while this was 29.9% in the group of respondents with a high educational level. This is in line with Verlinde et al., 2012, concluding that a lower preference for a shared decision making style is noticed in lower education people [13]. Patients with a higher educational level also tend to participate more in the consultation in terms of asking questions and asking for explanations and clarification than patients from a lower educational level. 

Regarding the respondents' test of preference, a considerable and statistically significant difference between hospitals A and E (Table 5) was observed. Next to the small differences in age distribution and influence of others, also provider beliefs and bias in counseling might have played a role.

 In this era of rapid developments in genetic testing and growing societal individualization, a uniform test policy seems rather out of date while, not only in healthcare, autonomy is increasing. From an ethical point of view, the best approach appears to be to offering women a choice between rapid targeted testing and karyotyping [14]. In a previous Dutch study of Boormans et al. on the impact of karyotyping and rapid targeted testing on quality of life, women showed a clear preference for rapid targeted testing or karyotyping. Despite individual differences, their study showed no systematic differences in time of stand-alone rapid targeted testing versus karyotyping in terms of anxiety, general physical and mental health, and stress [15]. 

 A limitation of our study is that participants provided the data after they had received their diagnostic test result. Thus, it is possible that attitudes were influenced by the stage of decision making and may have served, to some extent, as a post hoc justification for the choice. A second limitation is that the timespan between sampling and having the questionnaire varied between 4 and 28 months. It might be possible that respondents' attitudes and experiences have changed in time. This large timespan could also have influenced women's participation and might be an explanation of the low response rate (61.8%). 

 Although the group of respondents who received an abnormal test result was small (1.5%), women indicated to feel positive about having a test result quickly after they chose to have a rapid targeted test. Clearly, prospective studies are needed to examine this possible effect more clearly and examine choice of testing and likelihood of pregnancy termination.

 In comparison with rapid targeted testing, a slightly higher preference for karyotyping (52.7%) was indicated. A reason for this preference might be that karyotyping will detect all microscopically visible chromosomal abnormalities or respondents are already familiar with karyotyping. However, this result is not representative for the test preference in our daily practice. During the last year, the preference for rapid targeted testing was 60% versus 40% for karyotyping. 

 Rapid targeted tests have shown to improve cost-effectiveness as compared to karyotyping [16–18]. This appeared most beneficial when rapid targeted tests were applied as a replacement for karyotyping within larger laboratories (>1100 specimens per annum). However, including an individualized choice, assuming that 50% choose karyotyping and 50% a rapid targeted test, already has a major impact on costs [17]. While individual choice as a strategy is less efficient than a uniform strategy in which every patient would receive a rapid targeted test, an overall cost reduction remains in comparison with karyotyping [17]. 

 We conclude that respondents expressed a positive attitude towards the option to choose. The application of offering a choice in prenatal tests will, however, depend on preference of the prenatal centre and is mainly driven by sample throughput, overall costs, and the national health system concerned.

### 4.1. What's Already Known about This Topic?

- Offering an individualized choice in prenatal diagnosis is an appropriate strategy.

### 4.2. What Does This Study Add?

- The study gives us more insight in testing behavior and women's perceptions associated with having a choice between rapid targeted testing and karyotyping.
  - We revealed attitudes and experiences of women towards invasive prenatal diagnosis in case they were offered a real choice between a rapid targeted test and regular karyotyping.
  - Women expressed a positive attitude towards the option to choose.

## Figures and Tables

**Table 1:** Personal characteristics of the 825 respondents.

	Number	%
Highest educational level (*N* = 824)		
Lower vocational, lower secondary school	17	2.1
Intermediate and higher vocational, higher secondary school	348	42.2
College/university	459	55.7
Respondents per hospital (*N* = 825)		
A	292	35.4
B	187	22.7
C	94	11.4
D	96	11.6
E	156	18.9
Test of choice (*N* = 822) per hospital		
Karyotyping (overall)	433	52.7
A	184	63.0
B	112	59.9
C	37	39.4
D	50	52.1
E	50	32.7
Rapid targeted test (overall)	333	40.5
A	91	31.2
B	60	32.1
C	50	53.2
D	40	41.7
E	92	60.1
Both (overall)	10	1.2
Unknown (overall)	46	5.6

**Table 2:** Data about the general attitude of women about having the option to choose between prenatal tests.

	Strongly agree *N* (%)	Somewhat agree *N* (%)	Neither agree nor disagree *N* (%)	Somewhat disagree *N* (%)	Strongly disagree *N* (%)	Total *N* (%)
Do you agree that pregnant women should have the option to choose between tests.	628 (76.5)	176 (21.4)	7 (0.9)	6 (0.7)	4 (0.5)	821 (100)
Pregnant women are capable to choose between tests.	423 (51.6)	316 (38.5)	28 (3.4)	40 (4.9)	13 (1.6)	820 (100)
The choice can be best made by a professional.	45 (5.5)	226 (27.6)	105 (12.8)	335 (40.9)	109 (13.3)	820 (100)
Choosing between tests places a burden to pregnant women.	32 (4.1)	112 (14.1)	81 (10.3)	366 (46.3)	199 (25.2)	790 (100)

**Table 3:** Comments from the respondents who showed regret about their choice.

Case ID	Test of choice	Comment	Type of comment	Age	Hospital
59	Karyotyping	“There was too little amniotic fluid to perform karyotyping and therefore a rapid targeted test was the alternative test. I preferred karyotyping.”	Technical aspect	39	E
171	Karyotyping	“The option to choose was offered just before the procedure. I had my doubts and subsequently, I regret my decision.”	Personal aspect	39	B
266	Rapid targeted test	“Later in pregnancy a fetal heart defect was detected, possibly caused by a chromosomal aberration”	Medical aspect	40	B
463	Rapid targeted test	“Our test of choice was a rapid targeted test, contamination with blood made the test result unreliable. Unfortunately, we had to wait 3 weeks to obtain the karyotyping result”	Technical aspect	39	B
673	Karyotyping	“I will never make that choice again”	Personal aspect	33	A
725	Rapid targeted test	“Later in pregnancy a fetal heart defect was detected. We were uncertain whether this was caused by a chromosomal aberration”	Medical aspect	38	E
729	Unknown	“It went against all my feelings”	Personal aspect	40	A
745	Rapid targeted test	“In my previous pregnancy karyotyping was performed. It feels better when they examine more”	Personal aspect	38	E

**Table 4:** Data of personal experiences about the option to choose.

	Number	%
Do you regret the choice you made? (*N* = 823)		
No	811	98.5
Yes	8	1.0
Not applicable	1	0.1
Regret of invasive procedure	3	0.4
Did you make the decision freely? (*N* = 819)		
No	29	3.5
Yes	788	96.2
Not applicable	2	0.3
Did the opinion of others influence your choice? (*N* = 824)		
No	289	35.1
Yes, the professional	86	10.4
Yes, my partner	237	28.8
Yes, both professional and partner	163	19.8
Yes, both professional, partner, and others	49	5.9
Do you want to have the possibility to choose again in a next pregnancy? (*N* = 821)		
No, I prefer to have the choice made by the professional	22	2.7
Yes	795	96.8
Not applicable	4	0.5

**Table 5:** Characteristics of the respondents of the two hospitals A and E.

	Hospital A (%)	Hospital E (%)	*P*-value
Test of choice			<0.001
Rapid targeted test	31.2	59.5	
Karyotyping	63.0	33.3	
Education level			
Lower vocational, lower secondary school	1.1	0.6	
Intermediate and higher vocational, higher secondary school	41.4	42.6	
College/university	57.5	56.8	
Maternal age			0.004
<35	6.3	15.0	
≥36	93.7	85.0	
Regret			
No	99.3	98.1	
Influence			0.025
No	36.0	25.2	
Yes:	64.0	74.8	
(i) Professional	5.5	13.5	
(ii) Partner	39.3	21.9	
(iii) Professional and partner	12.0	32.9	
(iv) Professional, partner, and others	7.2	6.5	
